# Effect of Collaborative Governance on Medical and Nursing Service Combination: An Evaluation Based on Delphi and Entropy Method

**DOI:** 10.3390/healthcare9111456

**Published:** 2021-10-27

**Authors:** Beiquan Chang, Yansui Yang, Guillermo Andres Buitrago Leon, Yuzhong Lu

**Affiliations:** 1 School of Public Policy and Management, Tsinghua University, Beijing 100084, China; 2 Shenxiang School of Business, Sanda University, Shanghai 201209, China; guruclef82@outlook.com; 3 Glorious Sun School of Business, Donghua University, Shanghai 200051, China; sunny07111@126.com

**Keywords:** medical and nursing care combination, pension services, collaborative supply, index construction, evaluation of synergistic effect

## Abstract

[Background]: Improvement of synergies in medical and nursing services can help governments to optimize the allocation of medical resources; however, an appropriate evaluation method is critical for a suitable decision process in this regard. [Method]: To assess the medical and nursing service combination (MNSC) at a regional level, this study applied a five-dimension evaluation index composed of 28 basic response areas related to the MNSC development status in China, determining its respective weight through the Delphi and entropy methods. [Result]: This empirical exercise analyzed the MNSC supply system by interviewing nine heads of medical and nursing institutions and eleven healthcare-related government personnel during August of 2020 in Xinxiang City, Henan province, P.R China. Results showed: (1) public satisfaction with the fees charged by Medical and Nursing service Institutions (MNSI); (2) Medicare and supply services’ policy publicity; (3) the external financing situation of MNSI; (4) the medical staff’s professional quality; (5) the medical facilities and supply of MNSI; and (6) that the recognition level of the development plan of MNSI scored the highest effect on the synergy of MNSC supply among the assessed factors. [Conclusion]: These results showed that an evaluation based on the Delphi and entropy methods can effectively integrate the opinions of experts and related institutions to evaluate synergies on the medical and nursing service supply.

## 1. Introduction

Given the context of sustained population aging [1], providing appropriate healthcare support for the elderly is becoming increasingly challenging, especially because contradictions caused by the separation of medical care and nursing care are becoming increasingly prominent as the post-medical follow-up period is longer on senior ages, requiring sustained long-term professional care. The traditional healthcare model seems unable to provide an appropriate set of services as the separation between health and nursing care suggests that medical treatment and off-clinic eldercare is clearly defined, ignoring a comprehensive perspective as opportunities for synergies.

Although medical institutions can provide licenced treatment, chronic conditions, major diseases, and disabilities related to this segment require longer-term nursing support. Medical services may struggle to meet the daily needs associated with higher ages even more without the articulation of nursing service support in terms of prevention and illness identification; hospital services may experience case saturation with all problems associated with a “filled hospital beds” phenomenon. The Medical and Nursing Service Combination (MNSC) can effectively integrate prevention and treatment with emphasis on healthcare sustainability for senior citizens, thus addressing one of the main challenges that healthcare sectors around the world are trying to solve [2].

The World Health Organization (WHO) has called for health system reforms focused on comprehensive services that improve support regarding senior functional needs [3], as well as improvement in patient satisfaction, care perception of quality, and service access through integrated or coordinated comprehensive care [4]. In the case of China, despite going through notable developments in the last few decades, MNSC has started relatively late in contrast to developed countries in Europe and the United States and still has problems that require addressing. For example, the medical and nursing service system is still behind on service fragmentation and synergy development, and medical and nursing resource distribution presents a significant disparity between regions and institutions [5]. Further articulation of the Government, capital, hospitals, eldercare institutions, patients, and other related actors are fundamental for synergy development and service improvements within MNSC [6].

The MNSC collaborative supply refers to coordination and cooperation among subjects in a shared network to develop an interdependent and structured supply structure intended to maximize service satisfaction to ultimately increase public trust and confidence.

The Rainbow Model of Integrated Care (RMIC) is becoming internationally recognized for integrated care as a suitable theoretical framework [7] (Valentijn 2016). Although RMIC measurement tools have been used to assess coordination capabilities within certain diseases [7,8,9,10,11] there is still a need for an evaluation model at the regional level that meets the evaluation needs in the region analyzed in this exercise. Researchers have applied the theory of synergy on local and international scenarios [12,13] to study regional medical resources [14], public health services [15], the retirement pension service supply [16], and a combination of medical and nursing services [17]. However, existing research has mainly focused on the theoretical construction of collaborative supply mechanisms of combined medical care and eldercare services, with just a few studies on index system development to measure the synergy within MNSC in different regions from a practical view. As previously mentioned, a combined service supply may be the determining factor in increasing the life quality of the elderly population; likewise, a logical and scientifically supported evaluation mechanism on synergies for each specific context is urgently needed by both academia and industry to continue developing solutions at a theoretical and practical level.

Based on related literature about integrated care and senior health [18,19,20,21,22], in combination with the development status of integrated care in China [23], as well as healthcare professionals’ interviews based on the RMIC proposed framework, this study constructed an evaluation index system to assess the synergy effect of the MNSC service supply and applied it to the specific case of Xinxiang city, Henan province in China, whose findings provided useful information on MNSC synergy-related key factors, which were relevant and useful for local healthcare policy development.

The following sections of this paper explain: the construction of the mentioned evaluation index, describing its respective source and theoretical support (Section 2); the construction of the evaluation model for MNSC, including its index structure, weighting method, and calculation procedure (Section 3); the empirical application of the evaluation index, with its respective data source description, calculation process, and result interpretation (Section 4); and the respective conclusions and discussions of this study (Section 5).

## 2. Evaluation Index Construction for MNSC Supply

Based on the RMIC [9,24], there are three denominations for institutions that compose MNSC: (1) medical institutions within eldercare institutions [25]; (2) medical institutions that provide eldercare-related services [26]; and (3) institutions that provide medical and nursing services in accordance with a cooperation agreement between eldercare and medical care. In this last case, medical institutions send medical personnel to eldercare institutions on a regular basis to provide medical services, while at the same time establishing a green channel to facilitate access to medical treatment for senior citizens. Nursing services for rehabilitation and convalescence are the responsibility of the eldercare institution [26], resulting in a two-way referral care model [27,28].

### 2.1. Evaluation Index Theoretical Framework

As part of the theoretical base for this study, the RMIC model focuses on the identification of the MNSI four-factor core system: (1) person-centeredness; (2) service coordination; (3) professional coordination; and (4) organizational coordination. The identification of its respective four-factor auxiliary system consists of: (1) community centeredness; (2) technical competence; (3) cultural competence; and (4) system context (9), where these systems share a relationship of interdependence and mutual promotion. This interdependence can be also explained by the three main elements of the symbiosis theory [29]: (1) the symbiosis unit, related to the basic unit able to form a symbiotic relationship; (2) the symbiosis environment, defined by the sum of all the other elements, excluding the symbiosis unit; and (3) the symbiosis relationship, an interpretation that describes the benign and synergic element interaction exposing the nature of the formation and development of some systems [30,31,32].

MNSC embodies the symbiotic relationship between medical care and healthcare within an elder care context; in other words, it refers to the integration of medical resources and eldercare resources. This integrated model integrates disease diagnosis and treatment, rehabilitation care, and senior care services, breaking the traditional division between medical and caring institutions and driving integration effectiveness and resource utilization [33,34]. The introduction of the symbiosis theory into MNSC model research not only provides suitable theoretical support but can also play a positive role in the promotion of the integration of the medical care and healthcare systems.

The symbiosis elements also emerge in MNSC collaborative governance: (1) the symbiotic units can be described as the institutions on medical and caring systems, which, from a broader perspective, involves medical services and caring services with its related assets; (2) the symbiosis environment appears in the form of sectoral policies, standards, and norms appliable to MNSC; (3) the symbiotic relationship is precisely the object of study in the medical and care integrated service model exercise. Given its relevance in the Chinese context, the symbiotic environment has a special influence on the formation and development of the symbiotic relationship within the MNSC model.

Based on the compatibility between the symbiosis theory, the MNSC supply service model, symbiotic thinking, and the logic behind the rainbow model, it is possible to develop a theoretically supported and logically consistent evaluation method applicable to local level needs and relevant to researchers involved in pension model policy development.

### 2.2. Evaluation Index Construction for MNSC Supply

An evaluation index system that assesses the effects of the synergistic supply of medical and nursing services should allow for the integration of a theoretical basis with principles of scientific and reasonable evaluation index system construction, as well as the actual context and background of the MNSC development in China. This research shows that the collaborative development of the medical and nursing service system not only depends on the symbiotic units within the MNSC system and the supply of medical staff but also on the social environment and the stakeholder system of MNSC, where the symbiotic relation emerges [35,36,37,38]. Consistent with these elements, this study has developed an evaluation index for MNSC supply in China based on five dimensions: (1) the policy environment supply system; (2) the social environment supply system; (3) the professional network supply system; (4) and the medical care supply system and stakeholder system from the perspective of the synergetic theory. Details for each dimension are described as follows:

#### 2.2.1. Policy Environment Supply System

Mainly referring to relevant government functional departments and medical institutions involved in policy development and the resources environment, this group includes government departments, including the Health and Family Planning Commission, Office of Aging, Ministry of Civil Affairs, Ministry of Finance, Development and Reform Commission, and the Health Insurance Bureau [39]. In the Chinese context, these institutions usually possess strong authority within their own jurisdictions, and, although interdependent, they are also coordinated, with each department playing a clearly defined role in guiding the development and the application of MNCS supply [40]. This dimension considers their role in policy formulation, promotion, y implementation, and other related aspects of relevant government departments of MNSC in China.

#### 2.2.2. Social Environment Supply System

The social environment is defined by the surrounding environment for each MNSI, as the social support cannot be overseen in the process of service construction and development, since some related factors may influence the development of MNSI. Related factors include: (1) a relevant insurance system in combination with healthcare, since the development of eldercare and medical services in China is currently carried out through regulations and issuances from province-level regulations and policy documents [41]; (2) legal system related to MNSC, defined as the basic warranty for the development of MNSC; (3) market development mechanism construction—the development degree of MNSI and its market mechanisms are closely related [42]; (4) MNSI and its external financing situation, as MNSC requires constant financial support and funding constraints determine the pace and sustainability of institutional construction and expansion [43]; (5) the supervision mechanism for MNSI—the government as the main body of macro-control oversees the supervision of the whole process of the MNSC supply system, guaranteeing a stable operation of the project [44,45].

#### 2.2.3. Professional Network Supply System

This index refers to the grade of guarantee the eldercare environment provides, along with the facilities and equipment that ensure coverage of seniors’ security needs [46]. This study also evaluates the grade of synergy in the MNSI professional network supply system in China by combining: (1) the supply of basic materials and equipment, such as facilities; (2) the standardization of medical and nursing institutions; (3) the degree of information construction in MNSI; and (4) the management efficiency of MNSI.

#### 2.2.4. Medical Care Supply System

This index refers to specific service providers in MNSI, including doctors, nursing professionals, rehabilitation doctors, chefs, and staff for other related services. They generally have the knowledge and professional skills considered a direct embodiment of MNSI quality. In addition to perceiving the direct effects of policy implementation, they are also the core of the MNSC supply system [47]. This factor is determinant for the assurance of safety for the attending senior patients and impacts their quality of life. The current study included this element in the evaluation index for synergies within MNSI.

#### 2.2.5. Stakeholders’ System

This refers to stakeholders directly and indirectly related to MNCS that reflect the degree of development and the contextual situation of MNCS. Among various actors, it includes the elder community, seniors’ families, relevant experts, the public, and the media, mentioned collectively in this study as “the public”. Given the extent and complexity of their relationship, the stakeholder system is large and complex, their effect is less evident, and the level of dependency is limited.

As MNSC needs the support of the factors previously mentioned, its recognition and feedback provided by the public complete the overall picture. This actor plays an indispensable role in the promotion and development of MNSC [48].

Having explained the five-set dimensions within the index set for synergy assessment on eldercare MNSC in China, each first-level and second-level index has been described in Table 1 as follows:

## 3. Method: Evaluation Model Construction for MNSC Service Supply

### 3.1. Index Weight Calculation Method

To calculate the weight of the evaluation indicators, there are multiple methods, which can generally be divided into three categories: (1) qualitative evaluation; (2) quantitative assessment; (3) and qualitative and quantitative analysis. The first one belongs to subjective evaluation methods, where the results are related to the evaluator’s perspective and the inference and analysis reflect the lens of their knowledge and practical experience. This type of evaluation may be criticized by a lack of objective and reasonable scientific basis [49,50]. Quantitative evaluation is based on historical data related to the research object, as well as certain accurate data analysis software, and has strong objectivity when supported by a large amount of sample data [51]. The qualitative and quantitative method quantifies the subjective evaluation criteria and expresses them as numerical values. This method combines the advantages of qualitative and quantitative methods [52].

The selection of this methodological approach relies on the following reasons: 

(1) As an anonymous questionnaire survey method intended for experienced professionals in a certain field of knowledge, the Delphi technique utilizes a reiterative survey–feedback communication process where the researcher provides a questionnaire with its respective background materials and each piece of feedback allows for better comprehension of the information given by each expert [53,54]. Thanks to its anonymity (or non-disclosure), it allows for more independence on judgements, as well as better use of each expert’s knowledge and experience. Thus, this method has a certain degree of objectivity given a set of comprehensive opinions, along with practical and scientific support [55].

(2) The entropy method is an objective method able to determine each indicator’s weight based on the size of the information provided by the observed value of each indicator. This method avoids, to a certain extent, the defects of the subjective assignment method on weight calculation for each subsystem and index in this study, and greater values mean a greater incidence for the assessed index within the overall evaluation [56].

(3) Considering that MNSC in China is still in a developmental stage, it is difficult to collect enough objective data. Thus, an entirely quantitative method may not be entirely appropriate, and for this reason, a combination of both methods may provide stronger support and structure—on one side, the Delphi method effectively integrates the professionals’ expertise in the MNSC field, and on the other, the entropy method will calculate each respective weight effectively, avoiding the human factor-related deviation.

### 3.2. Index Weight Calculation Process

This empirical exercise utilized a five-level Likert questionnaire (for details please refer to attachment 1) where experts were asked to rate the importance of each index according to their own practical experience and theoretical knowledge, where 1 represents the lowest importance to (from 1 as the least importance to 5 as the most importance). With the collected data, after applying the reliability and validity tests, the entropy weighting method was used to calculate the weight value for each evaluation index. Details on the calculation process are described below.

First, under the assumption that the MNSC service supply is assessed through *m* index and *n* samples, the original data matrix $X={\left(x_{ij}\right)}_{m\times n}$ is standardized according to the following equation:

(1) $$x_{ij}{}^{\prime^{+}}=\frac{x_{ij}-\min x_{i}}{\max x_{i}-\min x_{i}}+1\;x_{ij}{}^{\prime^{-}}=\frac{\max x_{i}-x_{ij}}{\max x_{i}-\min x_{i}}+1$$

where $x_{ij}{}^{\prime^{+}}$ and $x_{ij}{}^{\prime^{-}}$ are respectively the positive and negative indicators; and $x_{ij}{}^{\prime }$ is the standardized value for *j*th index for the *i*th sample for i=1,2,…,n and j=1,2,…,m.

Secondly, the indicator proportion $P_{ij}$ is calculated for each *i* object under each *j* index as follows:

(2) $$P_{ij}=\frac{x_{ij}{}^{\prime }}{\sum_{i=1}^{n}x_{ij}{}^{\prime }}$$

Then, according to the definition of information/entropy [57], $E_{j}$ was calculated for *j*th index according to (3).

(3) $$E_{j}=-k\overset{n}{\underset{i=1}{\sum }}p_{ij}\ln\left(p_{ij}\right)$$

where k=1/ln(n) and $E_{j}\geq 0$. The difference coefficient $G_{j}$ is calculated as follows:

(4) $$G_{j}=\frac{1-E_{j}}{m-E_{e}}$$

where $E_{e}=\overset{m}{\underset{j=1}{\sum }}E_{j}$; $0\leq G_{j}\leq 1$;$\;\overset{m}{\underset{j=1}{\sum }}G_{j}=1$; a greater value means higher determinacy of the overall evaluation and a smaller entropy. Then, the weight of each evaluation index is calculated as follows:

(5) $$W_{j}=\frac{G_{j}}{\sum_{j=1}^{m}G_{j}}$$

### 3.3. Measurement Model Construction to Measure Synergy within MNSC Supply

The synergistic or coordination level of the MNSI supply system refers to the consistency degree among service supply subsystems: policy environment, social environment, professional network, and production and stakeholder among process development and improvement. This construction is based on the system synergy degree measurement model developed by [30], and the respective procedure is described as follows.

Firstly, the order degree for each subsystem is calculated as $S=\left\{S_{1},S_{2},S_{3},S_{4},S_{5}\right\}$ where $S_{i}\;i=1,\ldots ,5$ represents each index or service supply subsystem as follows:

$S_{1}$: Policy Environment
$S_{2}$: Social Environment
$S_{3}$: Professional network
$S_{4}$: Production
$S_{5}$: Stakeholder

For each subsystem, $S_{i}\;$, $e_{ij}$ represents the parameter corresponding to each subsystem where j=1,…,n, n≥1, $\beta_{ij}\leq e_{ij}\leq \alpha_{ij}$ being $\alpha_{ij}$ and $\beta_{ij}$ the upper and lower limits of the sequence parameter $e_{ij}$ at the system critical point.

In this study, the order parameter of each subsystem is the corresponding secondary evaluation index in the evaluation system of collaborative supply of MNSC in China. For example, the policy environment supply subsystem (*n* = 5), and its corresponding five secondary evaluation indexes are used as its order parameters $e_{ij}$, to obtain the system order degree $u_{i}\left(e_{ij}\right)$ according to (6) as follows:

(6) $$u_{i}\left(e_{ij}\right)=\{\begin{array}{c} \frac{e_{ij}-\beta_{ij}}{\alpha_{ij}-\beta_{ij}},\;j\in x_{ij}{}^{\prime^{+}}\\ \frac{\alpha_{ij}-e_{ij}}{\alpha_{ij}-\beta_{ij}},\;j\in x_{ij}{}^{\prime^{-}} \end{array}$$

where $u_{i}\left(e_{ij}\right)\in \left[0,1\right]$ and represents the contribution of the order parameter $e_{ij}$ to it respective subsystem. Higher values for $u_{i}\left(e_{ij}\right)$ imply a higher contribution.

The measure model of order degree for each $S_{i}$ subsystem $u_{i}\left(S_{i}\right)$ was constructed by linear weighting method as described as follows:

(7) $$u_{i}\left(S_{i}\right)=\overset{n}{\underset{j=1}{\sum }}w_{j}u_{i}(e_{ij}),w_{j}\geq 0,\overset{n}{\underset{j=1}{\sum }}w_{j}=1$$

where $w_{j}$ represents the weight of each order parameter. Higher values for $S_{i}$ mean a higher order of the subsystem $S_{i}$.

Assuming an initial order degree for the set of supply subsystems (policy environment, social environment, professional network, production, and stakeholder) in a $t_{0}$ moment: $u_{1}{}^{0}\left(S_{1}\right),\;u_{2}{}^{0}\left(S_{2}\right),\;u_{3}{}^{0}\left(S_{3}\right),\;u_{4}{}^{0}\left(S_{4}\right),\;u_{5}{}^{0}\left(S_{5}\right)$. Then, after a certain period, it will evolve to $t_{1}$ moment to a new order degree $u_{1}{}^{1}\left(S_{1}\right),\;u_{2}{}^{1}\left(S_{2}\right),\;u_{3}{}^{1}\left(S_{3}\right),\;u_{4}{}^{1}\left(S_{4}\right),\;u_{5}{}^{1}\left(S_{5}\right)$. Afterwards, the synergy of the system *SE* (coordination degree) is calculated as described in (8):

(8) $$SE=\theta \sqrt{\left|\overset{5}{\underset{i=1}{\prod }}\left[u_{i}{}^{1}\left(S_{i}\right)-u_{i}{}^{0}\left(S_{i}\right)\right]\right|}$$

where i∈{1,2,3,4,5}, θ∈[0,1], and

$$\theta =\frac{\underset{i}{min}\left[u_{i}{}^{1}\left(S_{i}\right)-u_{i}{}^{0}\left(S_{i}\right)\neq 0\right]}{\left|\underset{i}{min}\left[u_{i}{}^{1}\left(S_{i}\right)-u_{i}{}^{0}\left(S_{i}\right)\neq 0\right]\right|}$$

A higher value of *SE* means a higher degree of combination for MNSC supply in the investigated area. As the data collected for this study in this area of research has no change in time, the subsystem set of $t_{1}$ can be used for reference [7]. This empirical exercise defined the MNSC system service in China as described in (9) as follows:

(9) $$SE=\theta \sqrt{\left|\overset{5}{\underset{i=1}{\prod }}\left[u_{i}{}^{1}\left(S_{i}\right)\right]\right|}$$

where

(10) $$\theta =\frac{\underset{i}{min}\left[u_{i}{}^{1}\left(S_{i}\right)\right]}{\left|\underset{i}{min}\left[u_{i}{}^{1}\left(S_{i}\right)\right]\right|}$$

Finally, the grading definition for MNSC is based on actual literature [7,31,32], also considering expert suggestions, and interviewed personnel during the field investigation period. The respective grading standard for MNSC in China is described in Table 2:

## 4. Result: Empirical Analysis of Synergy Assessment of MNSI in China

### 4.1. Data Sources

#### 4.1.1. Index Evaluation

The study supporting the index set development for the relevant service subsystems applied a structured questionnaire (available in Supplementary Materials) to nine heads of MNSI and eleven healthcare-related government personnel during August of 2020. During the field investigation, the data collection instrument was applied personally to each one of the interviewed professionals. After data screening, 82.6% of the answers were valid. Details on the sample for the interview are described in Table 3.

#### 4.1.2. Empirical Analysis (Professionals)

Subsequently, the evaluation index set for the MNSC supply was empirically assessed by applying the specific questionnaire (available in Supplementary Materials) survey to managers, medical staff, and senior service customers in four MNSI in Xinxiang City (Henan Province, China). From 181 collected questionnaires, 172 were valid to an effectivity rate of 95%. Descriptive statistics are reported in Table 4.

#### 4.1.3. Empirical Analysis (Users)

To determine the evaluation given by the stakeholders to the MNSC synergy degree, an additional survey application (available in supplementary materials) was provided to the public as an online questionnaire through www.wjx.cn (accessed on 19 October 2021) [58] by obtaining a total of 245 responses that, after a validation check, resulted in 153 valid responses with an effectivity rate of 62.45%. Descriptive statistics are reported in Table 5.

### 4.2. Index Weight Determination

#### 4.2.1. Subsystem Order Degree and Synergistic Effect

Formulas (6) and (7) were applied to the sample data was used to define the contribution for each one of the five different perspectives: (1) healthcare government departments; (2) senior management personnel of MNSI; (3) medical personnel; (4) service users; and (5) the public; as well as the weight of the evaluation index for the effect of the collaborative supply of medical and nursing services. The order degree for each subsystem within the evaluation of synergies on MNSC supply was calculated and described in Table 6.

The synergistic degree of the composite system of MNSC supply is mainly affected by the order degree of each subsystem. As seen in Table 6, the order degree for (3) the professional network supply and (4) the medical care supply system is relatively high scoring, at 0.664 and 0.635, respectively. The order degree of the policy environment supply system and the social environment supply system is in the middle, at 0.60 and 0.521, respectively. The order degree of the stakeholder system is the lowest, at 0.496.

The parameter contribution also has an impact on the synergistic degree of its respective MNSC subsystem. As seen in Table 6, (4.3) professional quality of medical staff, (2.4) external financing of integrated care institutions, and (1.3) policy implementation in the promotion of MNSC, contribute around 0.3. However, a low contribution is also present in various cases, including in the: (5.4) fairness and accessibility of MNCS for the public’s enjoyment, which scored 0.029; (2.2) degree of legal system construction related to MNSC, at 0.033; (4.6) satisfaction of medical staff on their remuneration, at 0.036; and (5.1) public awareness of MNSI, at 0.040. These results indicate that the local government and MNSI would need to strengthen related supervision mechanisms, popularize the scope of the MNSI, recheck the salary calculation method and treatment of medical staff, and strengthen the publicity strategy of the MNSI among the public.

The order degree of each subsystem is influenced by its corresponding order parameter contribution degree. In the case of (1) policy supply system, the contribution of: (1.1) degree of construction of relevant policies of MNSC (0.63); (1.2) policy promotion of MNSC (0.65); and (1.4) support of government policies to medical and nursing institutions (0.68) have a total score that is less than (1.3) policy implementation in the promotion of MNSC (0.296). These results show that policy implementation has the greatest impact on the order degree of the mentioned supply subsystem.

In the social environment supply subsystem (0521), the contribution of (2.2) degree of legal system construction related to MNSC (0.033) scored the lowest, while (2.4) external financing of MNSI scored the highest (0.338). These results indicate that the external financing situation of the MNSI is determinant to the degree of synergy in this subsystem. Faced with the huge demand of eldercare in China, social capital is expected to be willing to be invested in the development of these institutions.

For the professional network supply subsystem (0.664), (3.6) satisfaction of senior population on MNSI contribution degree was relatively low (0.054), while the contribution degree of other order parameters was close to or above 0.1. To improve the order degree of the professional network supply system, it may be necessary to spend more resources on services for seniors, to improve their satisfaction levels.

In the medical supply subsystem association, (4.6) medical personnel salary satisfaction (0.036), (4.2) healthcare professionals, (4.1) availability of medical and support professionals (0.047) such as (4.4) medical staff training and examination mechanism (0.046), showed low levels of contribution, in contrast to (4.3) professional quality of medical staff (0.327), where the contribution was high and almost equal to that of the medical supply system. In this case, to enhance the order of the medical supply system, it may be necessary to improve multiple sub-variables such as the remuneration of medical staff, increase the supply of medical staff, establish an appropriate training and assessment mechanism for the medical staff within MNSI or other departments, improve the professional skills of medical staff, and improve their working environment.

Lastly, on the stakeholder supply subsystem, (5.4) fairness and accessibility of MNCS for the public’s enjoyment (0.029), (5.5) satisfaction of the public towards the waiting time to access MNSC (0.041), and (5.1) public awareness of MNSI (0.040) showed relatively lower contribution, whereas (5.6) public satisfaction with the fees charged by MNSI (0.164) showed the highest contribution. These results showed that, from the view of the main stakeholders’ subsystem, fee charges in public satisfaction are a key factor. To raise their satisfaction of stakeholders, institutions may need to increase public awareness and recognition through relevant knowledge diffusion, improve the public perception of fairness, and reduce the waiting time.

#### 4.2.2. Analysis of Supply System Synergy Degree

By including the calculation of the order degree for each subsystem in different regions into the Equation (9), it can be concluded that the degree of the synergy of the complex system of MNSC supply in the investigated region is 0.256, which is a low level of synergy. As mentioned previously, this value reflects that the synergistic supply of medical and nursing services is still in a primary developmental stage, having a less than evident synergistic supply effect not conducive to promoting the development of medical and nursing services in China. Therefore, the synergistic system of medical and nursing services in China has ample area for improvement.

Through the above-mentioned subsystem order analysis, it is apparent that the main reason for the low synergy of the combination of the medical and nursing service supply system is that the influence of the stakeholder system order is greater. Field research found that the policy promotion in various regions with regard to the medical and nursing service supply is not high, resulting in lower public awareness of the MNSI; in this research, there were individuals who had never even heard of medical and nursing integration—more so, various members of the public believe that the combination of medical and nursing integration for the general public is still far from a reality, given the difficulty in accessing such services. In addition, because the combination of healthcare is still in the initial stage of development in China, the construction of the relevant legal system is still being perfected.

## 5. Conclusions and Contributions

### 5.1. Research Conclusions

In the complex MNSC system of the Chinese MNSI, the goal of each subsystem is intended to contribute to the general service supply system. Based on the symbiosis theory, this study proposed a 5-dimension and 28-subdimension index evaluation system to assess the synergy degree of senior MNSC supply in the Chinese context. This study found that the mentioned evaluation system is composed of the following supply subsystems: (1) policy environment; (2) social environment; (3) professional network; (4) medical care; and (5) stakeholder system. To define this index structure, with a base in existing literature, this study was able to determine 28 second-level factors that may have an incidence on the Chinese MNSC supply.

Through the combination of the Delphi method and the entropy method, this study was able to consistently calculate weight values for each subsystem that composes the evaluation index set of synergy levels on the MNSC supply in China. By observing the obtained index weight results, it is possible to determine the following factors for consideration when attempting to increase the synergy level within MNSIs’ service supply: (1) the service charges of MNSI; (2) the implementation of government policies in the process of promoting the MNSC; (3) an active and effective social capital guide to the medical and nursing industry investment, ultimately to improve the external financing situation of the MNSI; (4) improve the professional quality of medical staff; and (5) strengthen the supply of medical facilities and medical staff in MNSI.

Based on the data obtained from the field survey, empirical assessment and software data analysis were used to determine the contribution degree for each order parameter and the overall synergy level of each subsystem to the MNCS supply in Xinxiang City. It was possible to conclude that that the order degree of the professional network supply system and the medical and nursing supply system was relatively high; the order degree of the policy environment supply system and the social environment supply system showed average values; while the order degree of the stakeholder system was the lowest. According to the collaborative supply effect of the MNSC survey section, the collaborative supply of MNSC in the Xinxiang city of China is still incipient in terms of collaborative form, and its collaborative supply effect is not evident and not conducive to promoting further development of MNSC in China.

### 5.2. Research Contributions

Current MNSC studies on a national and international research stream are mainly supported by the system contingency theory [59,60] and the coupling theory [61,62], among other similar theoretical frameworks [63]; however, studies on MNSC in China are relatively scarce. To contribute to filling this gap, this study applied the symbiosis theory to evaluate the coordination degree of MNSC supply by exploring the identified MNSC symbiosis core elements (professional network, medical staff, and healthcare stakeholders) and its correspondent symbiosis auxiliary elements (policy environment and social environment). Consequently, this research expands the application field and enriches the research perspective of the symbiosis theory in the specific area of MNSC in China.

Actual research on integrated care system evaluation mainly focuses either on assessing medical service provisions from an independent perspective [24] or the status of senior living service provisions [64]; however, there is not enough research on the combination of medical services and elderly care services, as well as the evaluation of integrated medical and elderly care service supplies. Based on the current context and objectives of the Chinese MNSC service supply, and from the symbiosis theory perspective, this study constructed a collaborative evaluation model for MNSC that may contribute effectively toward exploring improvement proposals regarding to the proposed object of study and supporting its analysis of MNSC.

This research not only provides a reasonable, scientifically supported model for the evaluation of MNSC supply but also contributes an applied empirical exercise by analyzing the specific case of MNSC supply in Xinxiang City (Henan Province), China and assessing the effect of the related key factors. The conclusions of this research may help policymakers to develop guidelines for an effective evaluation method on MNSC supply that can be further validated in different regions; it may also assist official organizations and MNSI to realize potential factors to promote the improvement of MNSC capabilities, as well as the integration and rationalization of resources to ultimately improve the life-quality of the senior population in China.

As this study’s empirical assessment is limited to the specific region of Xinxiang City, further research on a broader regional range and alternate samples will enrich the results presented in this paper.

## Figures and Tables

**Table 1:** Weight of evaluation index for collaborative supply effect on MNSC.

Primary Index	Weight	Secondary Index	Weight
1. Coordination of policy environment supply system	0.176	1.1 Degree of construction of relevant policies of MNSC	0.019
		1.2 Policy promotion of MNSC	0.019
		1.3 Policy implementation in the promotion of MNSC	0.090
		1.4 Support of government policies to medical and nursing institutions	0.018
		1.5 Relaxation and efficiency of government policy on the establishment of MNSI	0.030
2. Synergy degree of social environment supply system	0.144	2.1 How far is the insurance system related to MNSC?	0.019
		2.2 Degree of legal system construction related to MNSC	0.013
		2.3 Construction degree of market development mechanism on MNSC	0.019
		2.4 External financing of MNSI	0.075
		2.5. Construction of supervision mechanisms for MNSI	0.018
3. Collaboration of professional network supply system	0.212	3.1 What is the supply of facilities and equipment in MNSI?	0.057
		3.2 Standardization of MNSI	0.031
		3.3 Degree of informatization construction of MNSI	0.030
		3.4 Management efficiency of MNSI	0.045
		3.5 Convenience of the seniors to obtain medical care services	0.030
		3.6 Satisfaction of senior population on MNSI	0.019
4. Collaboration of degree of medical care supply system	0.174	4.1 Availability of medical and support professionals	0.018
		4.2 Medical staff professional skills	0.019
		4.3 Professional quality of medical staff	0.070
		4.4 Training and evaluation mechanism of medical staff	0.019
		4.5 Satisfaction of medical staff with their working environment	0.031
		4.6 Satisfaction of medical staff on their remuneration	0.017
5. Synergy degree of stakeholder system	0.295	5.1 Public awareness of MNSI	0.031
		5.2 Public recognition of the MNSC	0.045
		5.3 Public recognition of the MNSI development plan	0.057
		5.4 Fairness and accessibility of MNCS for the public’s enjoyment	0.017
		5.5 Satisfaction of the public towards the waiting time to access MNSC	0.031
		5.6 Public satisfaction with the fees charged by MNSI	0.113

**Table 2:** Grading definition for service collaboration.

Synergy Grade	*SE* Value	Interpretation
Optimal	0.8≤SE≤1	The service system shows the highest level of synergy, high efficiency is expected.
High	0.6≤SE<0.8	There is good collaborative effect and high synergy, the service supply system is expected to run effectively.
Average	0.4≤SE<0.6	The system collaborative effect is not significant, its synergy level has potential for further optimization.
Low	0.2≤SE≤0.4	The system collaborative effect is weak, its synergy is low and there is greater field for optimization initiatives.
Lowest	0≤SE≤0.2	The lowest synergy reflects a very weak collaborative effect among the service system, optimization is an evident need.

**Table 3:** Interviewed personnel descriptions.

Subject	Option	Quantity	Proportion
Work Unit	MNSC	8	42.11%
	Government related sector	11	57.89%
Gender	Male	12	63.16%
	Female	7	36.84%
Years Engaged in Position	Under 1 year	0	0%
	1–5 years	1	5.26%
	5–10 years	3	15.79%
	10–20 years	9	47.37%
	More than 20 years	6	31.58%
Age	Under 35 years of age	3	15.79%
	36–45 years of age	3	15.79%
	46–65 years of age	12	63.16%
	More than 65 years of age	1	5.26%

**Table 4:** Descriptive statistics for the empirical assessment of professional staff.

Subject	Option	Quantity	Proportion
Work Unit	MNSC	88	48.62%
	Government related sector	93	51.38%
Gender	Male	60	33.15%
	Female	121	66.85%
Post	Doctor	45	24.86%
	Nurse	60	33.15%
	Pension Personnel	59	32.60%
	Other	17	9.39%
Years Engaged in Position	Under 1 year	14	11.47%
	1–5 years	51	41.80%
	5–10 years	16	13.11%
	10–20 years	41	33.61%
	More than 20 years	43	23.76%
Age	Under 35 years of age	35	19.34%
	36–45 years of age	69	38.12%
	46–65 years of age	34	18.78%
	More than 65 years of age	88	48.62%

**Table 5:** Descriptive statistics for the empirical assessment of service users.

Subject	Option	Quantity	Proportion
Gender	Male	72	47.37%
	Female	80	52.63%
Age	Under 35 years old	46	30.26%
	36–45 years old	26	17.11%
	46–65 years old	77	50.66%
	More than 65 years old	3	1.97%
Highest degree	College and below	63	41.45%
	Undergraduate course	42	27.63%
	Master	28	18.42%
	Doctor	19	12.5%
Place of residence	Eastern China	95	62.5%
	Central China	57	37.5%

**Table 6:** Subsystem order degree in synergies on MNSC supply for this exercise.

Primary Index	Order Degree	Secondary Index	Parameter Contribution
1. Coordination of policy environment supply system	0.600	1.1 Degree of construction of relevant policies of MNSC	0.063
		1.2 Policy promotion of MNSC	0.065
		1.3 Policy implementation in the promotion of MNSC	0.296
		1.4 Support of government policies to medical and nursing institutions	0.068
		1.5 Relaxation and efficiency of government policy on the establishment of MNSI	0.108
2. Synergy degree of social environment supply system	0.521	2.1 How far is the insurance system related to MNSC?	0.051
		2.2 Degree of legal system construction related to MNSC	0.033
		2.3 Construction degree of market development mechanism on MNSC	0.049
		2.4 External financing of MNSI	0.338
		2.5. Construction of supervision mechanisms for MNSI	0.05
3. Collaboration of professional network supply system	0.664	3.1 What is the supply of facilities and equipment in MNSI?	0.051
		3.2 Standardization of MNSI	0.111
		3.3 Degree of informatization construction of MNSI	0.096
		3.4 Management efficiency of MNSI	0.156
		3.5 Convenience of the seniors to obtain medical care services	0.108
		3.6 Satisfaction of senior population on MNSI	0.054
4. Collaboration degree of medical care supply system	0.635	4.1 Availability of medical and support professionals	0.047
		4.2 Medical staff professional skills	0.090
		4.3 Professional quality of medical staff	0.327
		4.4 Training and evaluation mechanism of medical staff	0.046
		4.5 Satisfaction of medical staff with their working environment	0.089
		4.6 Satisfaction of medical staff on their remuneration	0.036
5. Synergy degree of stakeholder system	0.496	5.1 Public awareness of MNSI	0.040
		5.2 Public recognition of the MNSC	0.109
		5.3 Public recognition of the MNSI development plan	0.112
		5.4 Fairness and accessibility of MNCS for the public’s enjoyment	0.029
		5.5 Satisfaction of the public towards the waiting time to access MNSC	0.041
		5.6 Public satisfaction with the fees charged by MNSI	0.164

## Data Availability

Data used in this article comes from the specific questionnaire (available in Supplementary Materials) survey to managers, medical staff, and senior service customers in four MNSI in Xinxiang City (Henan Province, China), which were provided ethics approval and gave their consent to participate. The data of this study is confidential. If necessary, please contact the author.

## Supplementary Materials

The following are available online at https://www.mdpi.com/article/10.3390/healthcare9111456/s1, Supplementary Material 1: Expert Consultation Questionnaire (Determination of Weight) of Evaluation Index; System of Synergistic Effect of Combination of Medical Care and Supply; Supplementary Material 2: Questionnaire for persons in charge of integrated medical and nursing institutions and relevant government departments; Supplementary Material 3: Questionnaire of medical and nursing staff in medical and nursing institutions.

## References

[1] Chen R., Xu P., Song P., Wang M., He J. (2019). China has faster pace than Japan in population aging in next 25 years. Biosci. Trends.

[2] World Health Organization (2015). World Report on Ageing and Health.

[3] Briggs A.M., Valentijn P.P., Thiyagarajan J.A., De Carvalho I.A. (2018). Elements of integrated care approaches for older people: A review of reviews. BMJ Open.

[4] Baxter S., Johnson M., Chambers D., Sutton A., Goyder E., Booth A. (2018). The effects of integrated care: A systematic review of UK and international evidence. BMC Health Serv. Res..

[5] Wang W., Zhang T. (2016). Analysis of the relationship between medical care integration demand and medical resource allocation. Chin. Health Serv. Manag..

[6] Zhou Y., Li Y., Zhu X., Ma L. (2021). Medical and Old-Age Care Integration Model and Implementation of the Integrated Care of Older People (ICOPE) in China: Opportunities and Challenges. J. Nutr. Health Aging.

[7] Valentijn P.P. (2016). Rainbow of Chaos: A study into the Theory and Practice of Integrated Primary Care. Int. J. Integr. Care.

[8] Fares J., Chung K.S.K., Passey M., Longman J., Valentijn P.P. (2019). Exploring the psychometric properties of the Rainbow Model of Integrated Care measurement tool for care providers in Australia. BMJ Open.

[9] Valentijn P.P., Pereira F., Sterner C.W., Vrijhoef H., Ruwaard D., Hegbrant J., Strippoli G.F.M. (2019). Validation of the Rainbow Model of Integrated Care Measurement Tools (RMIC-MTs) in renal care for patient and care providers. PLoS ONE.

[10] Huang Y., Zhu P., Chen L., Wang X., Valentijn P. (2020). Validation of the care providers version of the Rainbow Model of Integrated Care-measurement tool in Chinese primary care systems. BMC Health Serv. Res..

[11] Wang X., Birch S., Chen L., Huang Y., Valentijn P. (2021). A Validation Study of the Rainbow Model of Integrated Care-Measurement Tool for Patients in China. Int. J. Integr. Care.

[12] Tulenko K., Møgedal S., Afzal M.M., Frymus D., Oshin A., Pate M., Quain E., Pinel A., Wynd S., Zodpey S. (2013). Community health workers for universal health-care coverage: From fragmentation to synergy. Bull. World Health Organ..

[13] Cassel J.B., Del Fabbro E., Arkenau T., Higginson I.J., Hurst S., Jansen L.A., Poklepovic A., Rid A., Rodón J., Strasser F. (2016). Phase I Cancer Trials and Palliative Care: Antagonism, Irrelevance, or Synergy?. J. Pain Symptom Manag..

[14] Wang J., Dai X., Yang Y., Ma Y. (2019). Effect of Collaborative Governance of Private Medical Institutions in Underdeveloped Counties of Central China: A Case Study of Macheng County in Hubei Province. Econ. Geogr..

[15] Wu S. (2019). Fragmentation and integration of public health service supply: A conceptual framework of collaborative governance. J. Guizhou Prov. Party Sch..

[16] Lu J., Liu W. (2017). Research on the Collaborative Supply of Community Elderly Services in Nanning City.

[17] Wang Z., Lu J. (2016). The Coordination Mechanism Research of Multiple Suppliers within Medical Serverices for the Old—Based on IDEA Strategic Model.

[18] Angelini L., Carrino S., Khaled O.A., Riva-Mossman S., Mugellini E. (2016). Senior Living Lab: An Ecological Approach to Foster Social Innovation in an Ageing Society. Future Internet.

[19] Gillespie S.M., Shah M., Wasserman E., Wood N.E., Wang H., Noyes K., Nelson D., Dozier A., McConnochie K.M. (2016). Reducing Emergency Department Utilization Through Engagement in Telemedicine by Senior Living Communities. Telemed. e-Health.

[20] Reisenwitz T.H. (2017). Exploring Senior Living Alternatives to Institutional Care: Differences between Residents and Non-residents. Glob. Bus. Rev..

[21] González-Ortiz L.G., Calciolari S., Goodwin N., Stein V. (2018). The Core Dimensions of Integrated Care: A Literature Review to Support the Development of a Comprehensive Framework for Implementing Integrated Care. Int. J. Integr. Care.

[22] Nicholson C., Hepworth J., Burridge L., Marley J., Jackson C. (2018). Translating the Elements of Health Governance for Integrated Care from Theory to Practice: A Case Study Approach. Int. J. Integr. Care.

[23] Liang D., Mei L., Chen Y., Zhou P., Yang X., Huang J. (2020). Building a People-Centred Integrated Care Model in Urban China: A Qualitative Study of the Health Reform in Luohu. Int. J. Integr. Care.

[24] Van Rensburg A.J., Fourie P. (2016). Health policy and integrated mental health care in the SADC region: Strategic clarification using the Rainbow Model. Int. J. Ment. Health Syst..

[25] Hu W. (2019). The Strategic Importance of the Institutional Reform Improving Medical-nursing Combined Service under the Background of Healthy China Strategy. Adm. Reform.

[26] Pei R. (2018). SWOT Analysis of Model of Pension Institution Setting in Medical Institution. Med. Soc..

[27] Deng D., Li Y. (2018). Integrating Old Age Care with Medical Service:Rational Institution, Imbalance between Supply and Demand and Innovative Solutions. J. Xinjiang Norm. Univ. (Ed. Philos. Soc. Sci.).

[28] Goodwin N. (2013). Understanding integrated care: A complex process, a fundamental principle. Int. J. Integr. Care.

[29] Hassell M.P., May R.M. (1975). Stability and Complexity in Model Ecosystems. J. Anim. Ecol..

[30] Rosenberg E., Zilber-Rosenberg I. (2011). Symbiosis and development: The hologenome concept. Birth Defects Res. Part C Embryo Today Rev..

[31] Aanen D.K., Eggleton P. (2017). Symbiogenesis: Beyond the endosymbiosis theory?. J. Theor. Biol..

[32] Luo N. (2017). Research on Shaanxi Province Medical Care Integration Model Based on Symbiosis Theory.

[33] He Q., Zhao R., Chen G. (2021). Discussion on the old-age service model based on the symbiosis theory in Sichuan rural areas with integrated medical care and elderly care. J. Jinling Inst. Technol. (Soc. Sci. Ed.).

[34] Zonneveld N., Driessen N., Stüssgen R.A.J., Minkman M.M.N. (2018). Values of Integrated Care: A Systematic Review. Int. J. Integr. Care.

[35] Suter E., Oelke N.D., Adair C.E., Armitage C.E. (2009). Ten Key Principles for Successful Health Systems Integration. Health Q..

[36] Amiel J.M., Pincus H.A. (2011). The medical home model: New opportunities for psychiatric services in the United States. Curr. Opin. Psychiatry.

[37] Hunter C.L., Goodie J.L. (2010). Operational and clinical components for integrated-collaborative behavioral healthcare in the patient-centered medical home. Fam. Syst. Health.

[38] Koufi V., Malamateniou F., Vassilacopoulos G. (2010). A system for the provision of medical diagnostic and treatment advice in home care environment. Pers. Ubiquitous Comput..

[39] Zhuang Y., Zhang T., Chen H. (2016). Study on Statcls Quo of National and Beijing Policies on Integrated Health and Social Care. Med. Soc..

[40] Wang S., Zhang Z., Sun W. (2013). The Mode and Path of the Combination of Medical Care and Care—Research Report on Promoting the Combination of Medical Care and Pension Services. Soc. Welf..

[41] Griffiths S. (2010). Integrating Traditional Chinese Medicine: Experiences from China. Australas. Med. J..

[42] Yin B., Ou S. (2016). Innovation and Practice: Exploration of the old-age care model in Chongqing A institution. Hum. Resour. Dev..

[43] Kwon S. (2008). Future of Long-Term Care Financing for the Elderly in Korea. J. Aging Soc. Policy.

[44] Zhang J., Cai Y. (2018). Medical disputes and mediation in China: Government and responsibility shifting. China Inf..

[45] Wu F., Sheng Y. (2019). Social support network, social support, self-efficacy, health-promoting behavior and healthy aging among older adults: A pathway analysis. Arch. Gerontol. Geriatr..

[46] Lei Y., Chungui L., Sen T. Community Medical Network (CMN): Architecture and implementation. Proceedings of the 2011 Global Mobile Congress (GMC).

[47] Wei Y., Zhang L. (2020). Analysis of the Influencing Factors on the Preferences of the Elderly for the Combination of Medical Care and Pension in Long-Term Care Facilities Based on the Andersen Model. Int. J. Environ. Res. Public Health.

[48] Xu J., Wang M., Yuan C., Guo Y. The Present Situation Analysis and Development Countermeasure of the New Mode of the Combination of Wisdom Pension and Medical Care. Proceedings of the 2019 International Conference on Robots & Intelligent System.

[49] Rahman M.S. (2016). The Advantages and Disadvantages of Using Qualitative and Quantitative Approaches and Methods in Language “Testing and Assessment” Research: A Literature Review. J. Educ. Learn..

[50] Peng L.V., Sun W., Zhang F. (2004). Combined Evaluation Method for Regional Innovative Ability.

[51] Huang J., Wu Y. (2011). Research on Evaluating the Value of Stock Investment Based on Combined Weight Method. High Performance Networking, Computing, and Communication Systems.

[52] Upjohn M., Attwood G., Lerotholi T., Pfeiffer D., Verheyen K. (2013). Quantitative versus qualitative approaches: A comparison of two research methods applied to identification of key health issues for working horses in Lesotho. Prev. Veter. Med..

[53] Moore P.G., Lingstone H.A., Turoff M. (1977). The Delphi Method: Techniques and Applications. J. R. Stat. Soc. Ser. A Gen..

[54] Chen Y. (2010). Research on the Application of Delphi Method, Cluster Analysis and Factor Analysis in People’s Livelihood Survey: A Case Study of Jianye District, Nanjing City. East China Econ. Manag..

[55] Perez V.L., Schuler R. (1982). The Delphi Method as a tool for information requirements specification. Inf. Manag..

[56] Ma Y., Wu Y., Wu B. (2015). Comprehensive Evaluation of Urbanization Sustainable Development in the Yangtze River Delta—Based on Entropy Method and Quadrant Diagram Method. Econ. Geogr..

[57] Contreras-Reyes J.E. (2021). Lerch distribution based on maximum nonsymmetric entropy principle: Application to Conway’s game of life cellular automaton. Chaos Solitons Fractals.

[58] Changsha Ranxing Information Technology Co. L (2006). Wenjuanxing. https://www.wjx.cn/.

[59] Wang C., Tang J., Tang S. (2021). Research on the Influencing Factors of the Quality of Medical Care-Integrated Elderly Care Services in Wuhan: A Comparative Analysis of Clear Set Qualitative Based on System Contingency Theory. Med. Soc..

[60] Hamann M. (2011). Towards a Contingency Theory of Corporate Planning: What Has Been Accomplished so Far?—Results of a Systematic Literature Review. SSRN Electron. J..

[61] Wang W., Cao H. (2021). Research on the Satisfaction of the Elderly Care Service in the Integrated Medical Care Institutions: Based on the Theory of Consumer Surplus. Bus. Econ..

[62] Cao Y., Zhou W., Chu T., Chang Y. (2019). Global Dynamics and Synchronization in a Duopoly Game with Bounded Rationality and Consumer Surplus. Int. J. Bifurc. Chaos.

[63] Sun Q., Zhou L. (2020). Research on the Realization Path of Medical Care Integration Based on Coupling Theory: Taking Zhengzhou City as an Example. Manag. Eng..

[64] Gillespie S.M., Wasserman E.B., Wood N.E., Wang H., Dozier A., Nelson D., McConnochie K.M., Shah M. (2019). High-Intensity Telemedicine Reduces Emergency Department Use by Older Adults With Dementia in Senior Living Communities. J. Am. Med. Dir. Assoc..

