# Supplementary material for: Effect of Collaborative Governance on Medical and Nursing Service Combination: An Evaluation Based on Delphi and Entropy Method
Source: Healthcare (Basel). 2021 Oct 27;9(11):1456. doi: 10.3390/healthcare9111456 (PMC8622114; doi:10.3390/healthcare9111456)
Supplement: Supplementary file 1 [file healthcare-09-01456-s001.zip › healthcare-1358649-supplementary.pdf]

Supplementary Material 1

Expert Consultation Questionnaire (Determination of Weight) of Evaluation Index

System of Synergistic Effect of Combination of Medical Care and Supply

Dear experts,

Hello! We are the research group of "Research on Collaborative Supply of Combination of Medical Care and Health Care", a postdoctoral fellow in Tsinghua University. On the basis of referring to the index system of relevant theories and methods of research on combination of medical care and health care at domestic and international level, and combining the opinions of consulting experts, we have summarized the evaluation index database of collaborative effect of combination of medical care and health care in China. In view of your rich experience and ability in this field, we sincerely invite you to grade the weight of evaluation indexes in the "*Evaluation Index System of Synergistic Effect of Combination of Medical Care and nursing supply*" designed by us, hoping to get your valuable opinions, thank you for your cooperation and support!

We promise that all the information in this questionnaire will only be used for academic research and subject research. For your personal information and answer content, we will keep strictly confidential.

Research Group on Collaborative Supply of Post-doctoral Medical Care and Pension Services in Tsinghua University

Index system evaluation table

This evaluation is intended for the setting of index weight. Please combine your professional knowledge, work experience and understanding of the coordinated supply of medical and nursing services, give your evaluation, opinions and suggestions for the index system structure and various candidate indexes. The index system of this consultation consists of policy environment supply system (including 5 secondary indexes), market environment supply system (including 5 secondary indexes), professional network supply system (including 6 secondary indicators), medical staff supply system (including 6 secondary indicators) and stakeholder supply system (including 6 secondary indicators), with a total of 5 primary indicators and 28 secondary indicators. Please score according to the importance of the evaluation index: 5 points for the most important, 4 points for important, 3 points for general, 2 points for non-important and 1 point for very unimportant.

**Primary Index**	**Serial number**	**Secondary Index**	**Mark**
**Policy environment supply system**	**1**	The degree of policy construction related to medical and nursing combination	

	**2**	Policy promotion in the process of promoting the combination of medical care and nursing	
	**3**	The implementation of policies in the process of integrating medical and nursing care	
	**4**	The support of government policies to medical and nursing institutions	
	**5**	Relaxation and efficiency of government policy on the establishment of medical and nursing institutions	
**Market environment supply system**	**6**	How far is the insurance system related to the combination of medical care and nursing?	
	**7**	The degree of legal system construction related to the combination of medical care and nursing	
	**8**	Construction degree of market development mechanism combining medical care with nursing care	
	**9**	External financing of medical and nursing institutions	
	**10**	Construction of supervision mechanism of medical and nursing institutions	
**Professional network supply system**	**11**	Facilities and equipment supply of medical and nursing institutions	
	**12**	Standardization degree of medical and nursing institutions	
	**13**	Information construction of medical and nursing institutions	
	**14**	Management efficiency of medical and nursing institutions	
	**15**	The convenience of elderly access to medical and nursing services	
	**16**	Satisfaction of the elderly in medical and nursing institutions	
**Medical staff supply system**	**17**	Supply of medical and other professionals	
	**18**	Professional skills of medical staff	
	**19**	Professional quality of medical staff	
	**20**	Is the training and assessment mechanism sound	
	**21**	Satisfaction of working environment of medical staff	
	**22**	Salary satisfaction of medical staff	
**Stakeholder supply system**	**23**	Public awareness of medical and nursing institutions	
	**24**	Public recognition of the development model of combination of medical and nursing care	
	**25**	The public's recognition of the development plan for the integration of medical and nursing care	
	**26**	The fairness and accessibility of the public to enjoy the integrated medical and nursing services	
	**27**	Satisfaction of the public with the length of waiting in line for medical care and nursing services	

	**28**	Public satisfaction with the fees charged by medical and nursing care institutions	

——Thank you again for your participation——

Supplementary Material 2

Questionnaire for persons in charge of integrated medical and nursing institutions and relevant government departments

Dear Managers,

Hello! We are carrying out a research on the evaluation of the collaborative supply effect of the elderly care services in the integrated medical and nursing institutions. The purpose is to determine the collaborative governance of the relevant policy subjects and market entities of the integrated medical and nursing institutions and provide the basis for the evaluation and optimization of the collaborative supply effect of the elderly care services of the combination of medical and nursing services in China. We look forward to your help and support. Please answer the questions in the questionnaire item by item according to the actual situation and the real idea. We promise that all the information filled in this questionnaire is only for academic research and for research purposes. We will keep your personal information and answers confidential.

Thank you very much for taking time out of your busy schedule to complete our questionnaire!

Research Group on Collaborative Supply of Post-doctoral Medical Care and Pension Services in Tsinghua University

Part I basic information

1. Your work unit is (_____).
   A. Integrated medical and nursing institution
   B. Government department.

2. Your gender is (_____)
   A. Male
   B. Female

Part II basic issues

According to your understanding and experience, please make a judgment on the following items. Answer notes: according to the setting of each topic, 5 - very high / good; 4 - relatively high / good; 3 - general weight; 2 - relatively low / poor; 1 - very low / poor.

Q1. What do you think is the degree of construction of relevant policies for the integration of medical and nursing care in China?

5-Very high	4-Relatively high	3-General	2-Relatively low	1-Very low

Q2. What do you think of the publicity and promotion of relevant policies on the combination of medical and nursing care in China?

5-Very high	4-Relatively high	3-General	2-Relatively low	1-Very low

Q3. Do you think that the implementation of relevant policies on the integration of medical and nursing care in China?

5-Very high	4-Relatively high	3-General	2-Relatively low	1-Very low

Q4. Do you think that the support of Chinese government policies to the institutions of combination of medical and nursing care?

5-Very high	4-Relatively high	3-General	2-Relatively low	1-Very low

Q5. Do you think that the government policy of our country has relaxed the intensity and setting efficiency of the medical and nursing institutions?

5-Very high	4-Relatively high	3-General	2-Relatively low	1-Very low

Q6. What do you think is the degree of insurance system construction related to the combination of medical and nursing care in China?

5-Very high	4-Relatively high	3-General	2-Relatively low	1-Very low

Q7. Do you think the legal system construction degree related to the combination of medical and nursing care in China?

5-Very high	4-Relatively high	3-General	2-Relatively low	1-Very low

Q8, do you think the market development mechanism construction degree related to the combination of medical and nursing in China?

5-Very high	4-Relatively high	3-General	2-Relatively low	1-Very low

Q9. Do you think the degree of supervision mechanism construction related to the combination of medical and nursing care in China?

5-Very high	4-Relatively high	3-General	2-Relatively low	1-Very low

Q10. What do you think about the situation of external financing for medical and nursing institutions in China?

5-Very high	4-Relatively high	3-General	2-Relatively low	1-Very low

Q11. Do you have any suggestions on the relevant policies and the management of government departments in China?

____

____

____

Q12. What suggestions do you have on the market environment of integrated medical and nursing institutions in China?

____

____

____

——Thank you again for your participation——

Supplementary Material 3

Questionnaire of medical and nursing staff in medical and nursing institutions

Dear medical personnel,

Hello! We are carrying out a research on the evaluation of the collaborative supply effect of elderly care services in medical and nursing institutions. The purpose of this survey is to determine the collaborative supply of medical and nursing institutions, to provide the basis for the evaluation and optimization of collaborative supply effect of elderly care services in China. We look forward to your help and support.

Please answer the questions in the questionnaire according to the actual situation and real ideas. We promise that all the information filled in this questionnaire is only for academic research and for research purposes. We will keep your personal information and answers confidential.

Thank you very much for taking time out of your busy schedule to complete our questionnaire!

Research Group on Collaborative Supply of Post-doctoral Medical Care and Pension Services in Tsinghua University

Part I basic information

Please give an objective answer to the following questions according to your actual situation.

Your company belongs to? (_____) A.

medical institution B. pension institution

2. Your gender is (_____).

A. Male
B. female

3. What is your position? (_____)

A. Doctor
B. Nurse
C. Senior Citizen
D. Other job types

4. How long have you been engaged in this occupation (_____)
(Note: this topic may not be suitable for you, it can be optional)

A. Less than 1 year
B. 1-5 years
C. 5-10 years
D. more than 10 years

5. Your age is (_____)

A. 35 years old and below
B. 36-45 years old
C. 46-65 years old
D. 65 years old and above

Part II basic issues According to your understanding, understanding and experience, please make a judgment on the following items.

Instructions:

Please answer according to the situation of each question, 5-very high / good / satisfied / sufficient; 4-relatively high / good / satisfied / sufficient; 3-general; 2-relatively low / poor / satisfied / sufficient; 1-very low / poor / satisfied / sufficient. (Note: some questions may not be suitable for you, please fill in and answer them as much as possible) Q1. Facilities and equipment supply in your organization?

5-Very high	4-Relatively high	3-General	2-Relatively low	1-Very low

Q2. How standardized is your organization?

5-Very high	4-Relatively high	3-General	2-Relatively low	1-Very low

Q3. What is the degree of information construction in your organization?

5-Very high	4-Relatively high	3-General	2-Relatively low	1-Very low

Q4. Management efficiency of your organization?

5-Very high	4-Relatively high	3-General	2-Relatively low	1-Very low

Q5. How convenient is the service for the elderly in your institution?

5-Very high	4-Relatively high	3-General	2-Relatively low	1-Very low

Q6. What do you think is the supply of medical staff in your institution?

5-Very high	4-Relatively high	3-General	2-Relatively low	1-Very low

Q7. What do you think is the professional skills of medical staff in your institution?

5-Very high	4-Relatively high	3-General	2-Relatively low	1-Very low

Q8. What do you think is the professional quality of medical staff in your institution?

5-Very high	4-Relatively high	3-General	2-Relatively low	1-Very low

Q9. What do you think about the training of medical staff in your institution?

5-Very high	4-Relatively high	3-General	2-Relatively low	1-Very low

Q10. Are you satisfied with your current working environment?

5-Very high	4-Relatively high	3-General	2-Relatively low	1-Very low

Q11. Are you satisfied with the current salary situation?

5-Very high	4-Relatively high	3-General	2-Relatively low	1-Very low

Q12. How satisfied are you with the service of the organization?

5-Very high	4-Relatively high	3-General	2-Relatively low	1-Very low

Q13. How satisfie[illegible] are you with the f[illegible] e setting?

5-Very high	4-Relatively high	3-General	2-Relatively low	1-Very low

Q14. What suggestions do you have for the management and service of our country's integrated medical and nursing institutions and staff?

____

____

____

——This is the end of the questionnaire. Thank you again for your participation——

Appendix 4 Questionnaire for the public

Dear public,

Hello! We are carrying out a research on the evaluation of the collaborative supply effect of elderly care services in medical and nursing institutions. The purpose of this research is to determine the situation of stakeholders of the combination of medical and nursing services and provide the basis for the evaluation and optimization of collaborative supply of elderly care services in China. We look forward to your help and support.

Please answer the questions in the questionnaire according to the actual situation and real ideas. We promise that all the information filled in this questionnaire is only for academic research and for research purposes. We will keep your personal information and answers confidential.

Thank you very much for taking time out of your busy schedule to complete our questionnaire!

Research Group on Collaborative Supply of Post-doctoral Medical Care and Pension Services in Tsinghua University

Part I Basic information

1. Your gender is (_____).

A. Male

B. Female

2. Your age is (_____).

A. 35 years old and below
B. 36-45 years old
C. 46-65 years old
D. 65 years old and above

3. What's your final education background.

A. Junior college or below
B. Undergraduate
C. Master
D. Doctor

4. Wich area do you live at? (_____)

A. East China
B. Central China
C. Western China

Part II Basic issues

According to your understanding and experience, please make a judgment on the following items. Note: please answer according to the situation of each topic, 5-very understanding / satisfied; 4-relatively understanding / satisfied; 3-general; 2-not understanding / dissatisfied; 1never heard / very dissatisfied.

Q1. Do you have a certain understanding of the combination of medical care and nursing?

5-Very high	4-Relatively high	3-General	2-Relatively low	1-Very low

Q2. How much do you agree with the mode of combination of medical and nursing care?

5-Very high	4-Relatively high	3-General	2-Relatively low	1-Very low

Q3. Are you satisfied with the development plan proposed by relevant government departments?

5-Very high	4-Relatively high	3-General	2-Relatively low	1-Very low

Q4. Are you satisfied with the fairness of the general public's access to medical and nursing services?

5-Very high	4-Relatively high	3-General	2-Relatively low	1-Very low

Q5. Are you satisfied with the length of waiting in line for medical and nursing services?

5-Very high	4-Relatively high	3-General	2-Relatively low	1-Very low

Q6. Are you satisfied with the setting of charges for medical and nursing institutions?

5-Very high	4-Relatively high	3-General	2-Relatively low	1-Very low

Q7. What expectations and suggestions do you have for the development of integrated medical and nursing institutions in China?

____

____

____

——Thank you again for your participation——
